# Randomized controlled pilot trial of naloxone‐on‐release to prevent post‐prison opioid overdose deaths

**DOI:** 10.1111/add.13668

**Published:** 2016-12-13

**Authors:** Mahesh K. B. Parmar, John Strang, Louise Choo, Angela M. Meade, Sheila M. Bird

**Affiliations:** ^1^ MRC Clinical Trials Unit at University College LondonLondonUK; ^2^ National Addiction Centre at King's College LondonLondonUK; ^3^ MRC Biostatistics UnitUniversity of Cambridge Institute of Public HealthCambridgeUK

**Keywords:** Fatality, naloxone, opioid‐overdose, post‐release, prevention, prisoners, randomization, trial

## Abstract

**Background and Aims:**

Naloxone is an opioid antagonist used for emergency resuscitation following opioid overdose. Prisoners with a history of heroin injection have a high risk of drug‐related death soon after release from prison. The NALoxone InVEstigation (N‐ALIVE) pilot trial (ISRCTN34044390) tested feasibility measures for randomized provision of naloxone‐on‐release (NOR) to eligible prisoners in England.

**Design.:**

Parallel‐group randomized controlled pilot trial.

**Setting:**

English prisons.

**Participants:**

A total of 1685 adult heroin injectors, incarcerated for at least 7 days pre‐randomization, release due within 3 months and more than 6 months since previous N‐ALIVE release.

**Intervention:**

Using 1 : 1 minimization, prisoners were randomized to receive on release a pack containing either a single ‘rescue’ injection of naloxone or a control pack with no syringe.

**Measurements:**

Key feasibility outcomes were tested against prior expectations: on participation (14 English prisons; 2800 prisoners), consent (75% for randomization), returned prisoner self‐questionnaires (RPSQs: 207), NOR‐carriage (75% in first 4 weeks) and overdose presence (80%).

**Findings:**

Prisons (16) and prisoners (1685) were willing to participate [consent rate, 95% confidence interval (CI) = 70–74%]; 218 RPSQs were received; NOR‐carriage (95% CI = 63–79%) and overdose presence (95% CI = 75–84%) were as expected. We randomized 842 to NOR and 843 to control during 30 months but stopped early, because only one‐third of NOR administrations were to the ex‐prisoner. Nine deaths within 12 weeks of release were registered for 1557 randomized participants released before 9 December 2014.

**Conclusions:**

Large randomized trials are feasible with prison populations. Provision of take‐home emergency naloxone prior to prison release may be a life‐saving interim measure to prevent heroin overdose deaths among ex‐prisoners and the wider population.

## Introduction

Prisoners with a history of heroin injection have a high risk of drug‐related death (DRD) soon after prison release, which was estimated at five DRDs per 1000 eligible releases on the basis of record‐linkage studies in Scotland in 1996–99 1 and in England and Wales in 1999–2002 2; also see meta‐analyses 3, 4.

Naloxone is an opioid antagonist that can be administered intramuscularly and is used by emergency services to reverse heroin/opioid overdose 5. The feasibility of randomized provision of naloxone‐on‐release (NOR) to a high‐DRD risk population, such as inmates with a history of heroin injection use on their release from prison, as proposed by Bird & Hutchinson 1, had not been investigated. Prison‐based randomized trials must address either a concern that applies specifically to prisoners (as here) to counter the challenge that the same trial could have been conducted equally well in the outside community or be able to point to parallel trials on the outside to answer the challenge of exploiting prisoners’ captivity.

One recent international review of non‐randomized community initiatives on take‐home naloxone, mainly in the United States and the United Kingdom, with follow‐up for 3–6 months of their trainees, gave an estimated fatality rate at witnessed opioid overdose of 6% (upper 95% confidence limit = 11%), suggesting that a target for the annual distribution of naloxone kits should be nine to 20 times a nation's annual number of opioid‐related deaths 6. A recent comprehensive monograph 7 has documented the historical development (from the 1990s) and spread of take‐home naloxone programmes through North America, Europe and Australia and considered their practical implementation, including the training of naloxone recipients in how to recognize and respond to an overdose. Although supported by the World Health Organization (WHO) 8, barriers remain to accessing take‐home naloxone: in most European jurisdictions, naloxone is a prescription‐only medicine; in others (see below), its addition to the exempt list of prescription‐only medicines did little to change clinical practice, take‐home naloxone being deemed contentious 9 by some, complex 10 by others. When the notification of overdose events triggers a report to the police, this may discourage witnesses from contacting emergency medical services, and the need to inject naloxone can prove a psychological barrier for some responders as well as being a potential health risk for all who administer the injection 7.

In 2005, naloxone was added to the UK's exempt list of prescription‐only medicines that can be administered by anyone to save life in an emergency 11, 12. Bird *et al.* then applied to the UK Medical Research Council (MRC) for funding to conduct a randomized effectiveness study of NOR for prisoners with a history of heroin use by injection. The definitive NALoxone InVEstigation (N‐ALIVE) trial was to investigate if NOR could reduce DRDs in the first 4 weeks after release by 30% and in weeks 5–12 by 20%. In 2008, the Medical Research Council (MRC) funded the N‐ALIVE pilot trial to randomize the first tenth of 56 000 prisoners needed for the main trial: half in Scotland, the other half in 15 prisons in England and Wales. The rationale for the N**‐**ALIVE trial has been described previously 9.

In January 2011, Scotland became the first nation to make both community‐based take‐home naloxone and NOR for eligible prisoners a public health‐funded policy 6, 13, 14, 15. Wales followed suit later in 2011 16. Accordingly, N‐ALIVE was conducted in English prisons only, and its target accrual reduced to 2800 participants.

Our a priori estimation of the probable effectiveness of naloxone 1 accounted for someone else being present at four‐fifths of opiate overdoses 12; and that, most often, the others present (peers or family‐members) were willing to intervene but lacked effective means 17.

We report the key feasibility outcomes of the N‐ALIVE pilot trial, compared with prior expectations in the trial protocol (ISRCTN34044390) 18, as follows:

- participation by prisons and prisoners;
- consents for randomization, returned prisoner self‐questionnaire (RPSQ), telephone‐contact substudy;
- receipt of RPSQs;
- NOR carriage and overdose presence; and
- whether the N‐ALIVE main trial could go ahead as planned, including assessment of to whom NOR was administered.

## Methods

### Design: plausible effectiveness and main trial size, plus key assumptions to be checked by pilot trial

**Figure 1:**
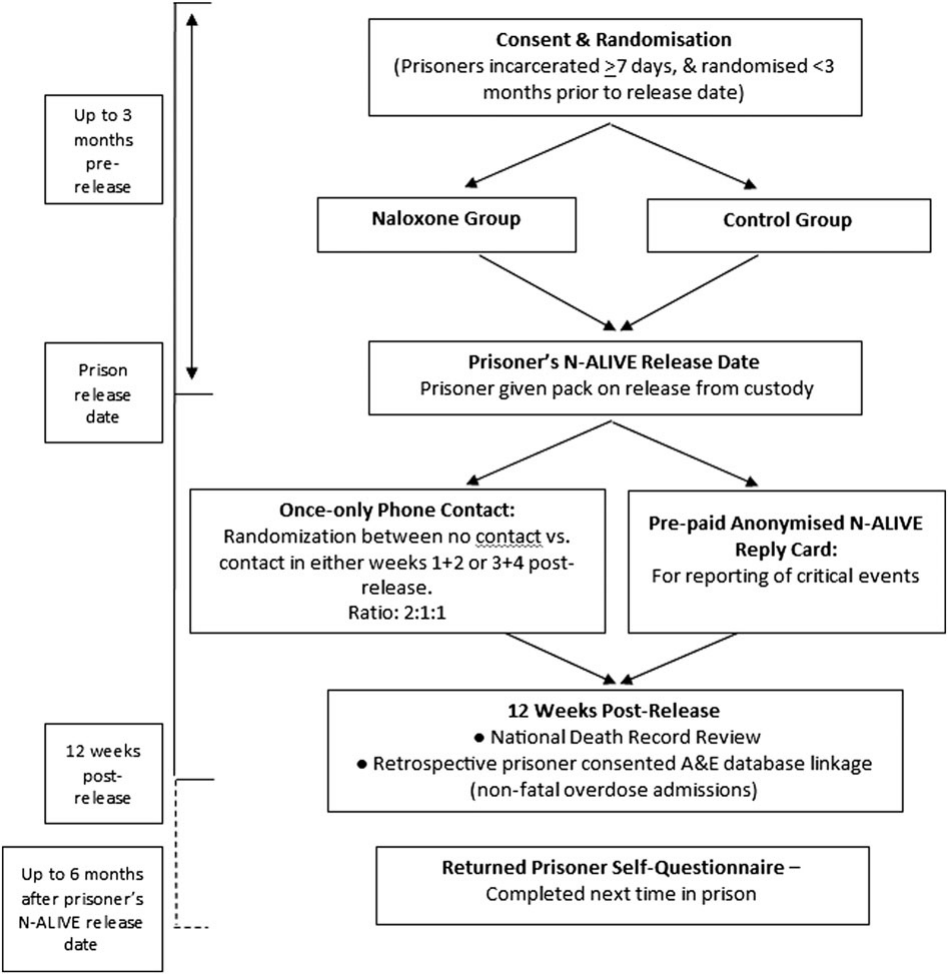
NALoxone InVEstigation (N‐ALIVE) pilot trial design and outcome measures. A full list of the trial's outcome measures is described in the N‐ALIVE protocol, which is available on the N‐ALIVE webpage: http://www.ctu.mrc.ac.uk/13391/13399/18277/n‐alive_trial_protocol

N‐ALIVE was a randomized controlled trial of parallel groups (see Fig. 1). Research‐trained prison‐based N‐ALIVE workers recruited and consented eligible prisoners.

The N‐ALIVE main trial was to investigate if NOR could reduce DRDs in the first 4 weeks after release by 30%, from 140 to 98 per 28 000 eligible releases; and in weeks 5–12 by 20%, from 35 to 28 per 28 000 eligible releases, for which randomization of 56 000 eligible releases would be needed for 80% power at a 5% significance level. In 2008, MRC funded the N‐ALIVE pilot trial to randomize the first tenth of 56 000 prisoners needed for the main trial: half in Scotland, the other half in prisons in England and Wales.

Our a priori estimation of NOR's probable effectiveness in the main trial took into account that: (a) someone else is present at 80% of opiate overdoses 12; (b) 75% of ex‐prisoners randomized to NOR would carry NOR in the first 4 weeks, reducing to 50% in weeks 5–12, but negligible thereafter 18; (c) most often, the others present (peers or family members) are willing to intervene 17 but, conservatively, we assumed a 50 : 50 chance that others present would have the presence of mind to locate, assemble and administer NOR to the ex‐prisoner 18. Hence, NOR's plausible effectiveness at reducing DRDs was 80 × 75 × 50%, or 30%, in the first 4 weeks, but reduced to 20% in weeks 5–12.

In Scotland and in England and Wales, the N‐ALIVE pilot trial was to test our key assumptions on (i) participation, (ii) consents, (iii) receipt of RPSQs, (iv) NOR carriage and overdose presence, and hence (v) whether the N‐ALIVE main trial could go ahead as planned, including by assessment of to whom NOR was administered.

Because the N‐ALIVE pilot trial could randomize in English prisons only, its target accrual reduced to 2800 participants, sufficient for assessing (i)–(v) for England. Key outcome measures and prior expectations for the pilot trial 18 are detailed in Table 1. The N‐ALIVE pilot trial was approved by Essex 2 Research Ethics Committee.

**Table 1:** Feasibility outcomes: summary of prior assumptions and actual findings.

Outcome	Prior assumption	Actual	Comment
Number of participating prisons	11 prisons in Scotland 14 prisons in England	16 prisons in England (15 open, 1 closed to recruitment)	Consistent with expectation
Target participant accrual	5600 participants Original sample size requirement was up to 10% of 56 000 participants	Target revised down to 2800 for England due to non‐participation of prisons in Scotland and Wales February 2014: interim target revised to 1500 by 31 August 2014 Actual accrual at 31 August 2014 was 1392 participants 1500 participants achieved on 8 October 2014 Final accrual at 8 December 2014: 1685 participants	Early cessation, see Fig. 3
Consent for randomization by eligible prisoners	75%	Based on screening logs, the consent‐rate for randomization among eligible prisoners was 72% (1283 of 1777); 95% CI = 70–74%	Upper 95% confidence limit is just short of our prior expectation
Consent to returned prisoner self‐questionnaire (RPSQ)	Prior expectation of 75%	Consent to complete the RPSQ was given by 85% of participants (1417 of 1676); 95% CI = 83–86%	Above expectation
Consent to secondary randomization in the telephone‐contact ancillary study	Prior assumption of 50%	Consent to take part in the telephone contact study was provided by 56% (946 of 1676); 95% CI = 54–59%	Above expectation
Number of RPSQs	333 recidivist self‐questionnaires expected from 2500 randomized and released participants, so we expected 333 of 2500 × 1557 = 207 RPSQs	218 received from 1557 randomized and released participants	Consistent with expectation
Carriage rate in first 4 weeks after release	75%	RPSQ 71% (80 of 112) 95% CI = 63–79%	Consistent with expectation
Someone else present at overdose	80%	Based on RPSQs: 53 of 205 recidivists (26%; 95% CI = 20–32%) of recidivists reported having injected when alone, and had done so on a mean of 6 of 14 days. Hence, 95% CI for someone else present is 68–80% Telephone questionnaire: heroin use in the past 3 days was reported by 31 of 81 telephone contacts (38%), 10 of whom had injected when alone (12%; 95% CI = 5–20%). If the past 3‐day rate is taken as representative of the rate throughout the first 4 weeks, then 95% CI for someone else being present at injector's overdose is 80–95% Both consistent with our prior expectation that someone else is present at 80% of opiate overdoses Pooled estimate (based on weights 61% and 39%) is 79%; 95% CI = 75–84%	Consistent with expectation
Telephone questionnaire phone contacts in the first or second fortnight	Based on the (probability of consent) × (probability of being randomized to telephone contact given consent) × (probability of contact given randomized to telephone contact) = 1/8 × number randomized and released (1557) = 195	81 of 1557 randomized and released participants Poisson 95% CI = 63–99	Well below expectation
Drug‐related deaths in first 4 weeks and next 8 weeks after release	We expect 1/200 × 1557 = 7.9 or 8 DRDs in first 4 weeks after release if NOR is not effective; and a further 1/800 × 1557 = 1.9 or 2 DRDs in the next 8 weeks	2 DRDs in first 4 weeks post‐release; a further 3 DRDs in the next 8 weeks were registered with Office for National Statistics by 21 April 2016 First 4 weeks, 95% CI = 0.2–7.2 12 weeks, 95% CI = 1.6–11.7	Below expectation for the first 4 weeks; consistent with expectation in the first 12 weeks
Non‐fatal overdose‐related admissions within 12 weeks of release	We assume participants’ non‐fatal overdose admissions to Accident and Emergency within 12 weeks of index release to be between two and eight times as many as DRDs with 2–3 times as many DRDs being our best estimate, thus we expect 20–30 (but up to 80) non‐fatal overdose‐related Accident and Emergency admissions	Awaiting Hospital Episode Statistics data from Health and Social Care Information Centre (now NHS Digital)	No information

CI = confidence interval; DRD = drug‐related deaths; NOR = naloxone‐on‐release.

### Data collection

Study forms were sent by our prison‐based N‐ALIVE workers to MRC Clinical Trials Unit by post or fax. Reporting of release date was particularly important as marking the start of a participant's at‐risk period. Screening logs for eligibility were introduced in September 2012.

The randomization form checked a potential participant's eligibility and consents and provided the information needed for minimization (see below). Other forms recorded the participant's release date or date of prison transfer. Information on date and cause of death within 4 weeks, 12 weeks and 6 months of a participant's N‐ALIVE release date was obtained from the Office for National Statistics by checking periodically against registered deaths, most recently on 21 April 2016. The RPSQ was designed to answer objectives (iv) and (v).

### Eligibility, randomization and consent

Eligibility criteria were age greater than 18 years (upper limit of 44 removed 16 months into the trial because 10% of otherwise eligible prisoners were being excluded, see Table 2), history of heroin use by injection, incarcerated for at least 7 days, expected release date within 3 months of randomization date, not previously randomized in N‐ALIVE trial and then consent withdrawn prior to release, and written informed consent. Exclusion criteria were known history of anaphylactic reaction to naloxone, confirmed/declared pregnancy or pregnancy intended within 6 months, normally resident outside the United Kingdom, and randomization date within 6 months of most recent N‐ALIVE release‐date (or, if missing, within 1 year of previous randomization date).

**Table 2:** Baseline characteristics for 1557 participants randomized and released by 8 December, 2014.

Characteristic	NOR		Control		All	
Age (Mean SD) years	Mean 35 years SD 7 years		Mean 35 years SD 6 years		Mean 35 years SD 7 years	
	*n*	%	*n*	%	*n*	%
Age categories (*n*, %) years						
18–24	40	5	40	5	80	5
25–34	381	50	385	50	766	50
35–44	290	38	302	39	592	39
45+	47	6	46	6	93	6
Gender (*n*, %)						
Males	762	98	771	98	1533	98
Females	12	2	12	2	24	2
Treatment for addiction (at randomization) (*n*, %)						
Opiate substitution	496	64	503	64	999	64
Opiate detoxification	163	21	163	21	326	21
Other (e.g. naltrexone, no current treatment)	114	15	116	15	230	15
Not recorded	1	<1	1	<1	2	<1
Probable incarceration interval (*n*, %)						
(Date of randomization–expected release date at randomization)						
Within 28 days	532	69	540	69	1072	69
4–12 weeks	171	22	175	22	346	22
>12 weeks	17	2	13	2	30	2
Unknown release date	54	7	55	7	109	7

SD = standard deviation.

NOR = naloxone‐on‐release

Participants were randomized (1:1) by the MRC Clinical Trials Unit to receive on release a pack containing either a single ‘rescue’ injection of naloxone or a control pack which did not contain naloxone—there was no placebo. Minimization (with 80 : 20 randomization) was applied across gender, age group (18–24, 25–34, 35+ years), re‐randomization, management of opioid dependency at randomization (substitution, detoxification, other) and probable interval from randomization to index release (within 28 days, 4–12 weeks).

Consent for mortality follow‐up was mandatory at the time of consent for randomization, but could be withdrawn while participants were still in prison: in such a case, the participant would be withdrawn from the trial and no attempt would be made to provide a pack on release. Additionally, prisoners were asked for their consent for (i) record‐linkage to establish if participants had had any admissions to accident and emergency departments for non‐fatal overdose in the 12 weeks following their N‐ALIVE release‐date; and (ii) randomization in a once‐only telephone contact substudy 18,; see Fig. 1 and Supporting Information.

The trial was double‐blind prior to release so that, while the participant was still in custody and pre‐release, neither the participant, prison‐based N‐ALIVE staff nor prison staff knew the allocation. Participants learned their allocation when they opened the pack at the time of their release.

### N‐ALIVE packs

Each prison had a supply of pre‐numbered sealed N‐ALIVE packs (Meade *et al*., in review). Control and naloxone packs were identical in appearance, sounded alike when shaken and were similar weights. All packs contained the N‐ALIVE DVD, a wallet and had tamper‐evident stickers attached.

The wallet in the control pack did not contain a syringe. The wallet in the naloxone pack included all the same material as the control wallet, but also contained a pre‐filled syringe, the unscrewed plunger rod for the syringe and a safety‐covered, sterile‐packed hypodermic needle; see Supporting information. The plunger had been removed from the syringe barrel so that both fitted into the N‐ALIVE wallet; both had to be fitted to the syringe before use. The syringe contained 2 mg of naloxone hydrochloride in 2 ml of solution, for once‐only intramuscular injection in the event of overdose. During information and consent sessions, participants were advised on how to administer the N‐ALIVE‐recommended 0.8 mg intramuscular dose of naloxone. For trial purposes, the naloxone needed to be a single product (see Meade *et al*., in review). As none of the available products fitted our needs well, the correct dose in single product form was most crucial. Accordingly, we selected the 2‐mg pre‐loaded syringe as an acceptable formulation for the pilot trial period (see Meade *et al*. in review), which necessitated additional instructions for administration to be limited to a 0.8 mg dose.

### Returned prisoner self‐questionnaire

We asked participants if they were willing to complete an anonymous follow‐up questionnaire if they returned to prison within 6 months of their most recent N‐ALIVE release date. The RPSQ identified the participant's randomized assignment and the time interval between the preceding N‐ALIVE release date and completion date but, to encourage frankness, the identity of the respondent was not recorded. Forms were neither checked nor overseen by the N‐ALIVE worker unless the respondent so chose.

### Statistical analysis

All validly randomized participants who were released from custody before 9 December 2014 are included in the analyses.

Simple summary statistics for percentages or counts [together with 95% confidence intervals (CIs)] are used to assess consistency with prior expectations: on consents, RPSQs, NOR carriage in the first 4 weeks, overdose presence and the use made of NOR to save the life of others than those for whom it was prescribed. Comparison of rates between NOR versus control group, based on RPSQs, is by χ^2^ or Fisher's exact test, as appropriate.

Formal comparison of risk behaviours versus perception between NOR versus control is based on a composite score for risk behaviours, derived *post hoc* from RPSQs. Early cessation of randomization in the N‐ALIVE pilot trial made it unlikely that subsequent NOR evaluations would be randomized individually. We therefore needed to make best use of the N‐ALIVE pilot trial's data to explore whether those randomized to NOR had increased the riskiness of their heroin use soon after release, as distinct from how participants perceived that their behaviour had been changed by taking part in N‐ALIVE.

### Risk score

To analyse how provision of NOR impacted on participants’ heroin use and related risk behaviours soon after release, a risk score based on RPSQ responses was devised by S.M.B. and A.M.M. and agreed by J.S. and M.K.B.P. (see Supporting information for how individual questions were scored), before being implemented by L.C. and tested for interaction (NOR versus control) against perceived behaviour change.

## Results

### Recruitment: prisons and participants

The trial was conducted in 16 prisons in England. Based on screening logs, the consent rate for randomization among eligible prisoners was 72% (1283 of 1777, 95% CI = 70–74%), just short of our prior assumption of 75% (Table 1).

**Figure 2:**
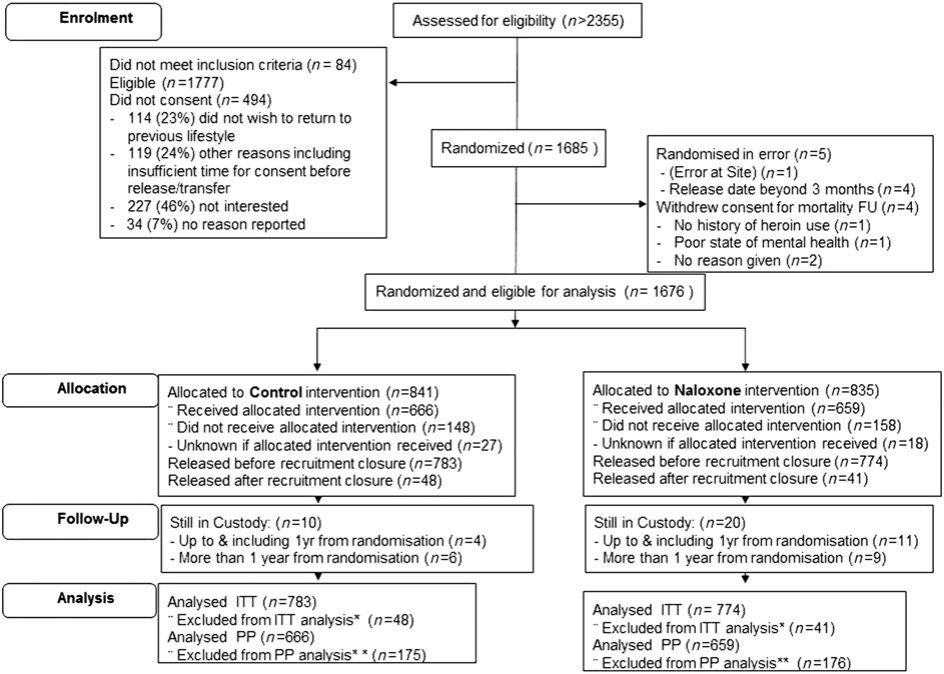
Consolidated Standards Of Reporting Trials (CONSORT) Diagram for the NALoxone InVEstigation (N‐ALIVE) pilot Trial. Screening records have been kept only since September 2012 to provide a snapshot of the proportions deemed eligible and subsequently randomized. *Excluded from intention‐to‐treat (ITT) analysis participants released after recruitment closure (*n* = 48, 40). **Included in per‐protocol (PP) analysis participants released with pack only

Between 28 May 2012 and 8 December 2014, we randomized 1685 participants (842 to NOR; 843 to control). Nine participants are considered ‘not randomized’ because they were withdrawn prior to release: four withdrew consent for mortality follow‐up prior to their release, while five were found to be ineligible, see Fig. 2 [Consolidated Standards Of Reporting Trials (CONSORT)].

Consent to complete the RPSQ was given by 85% of participants (1417 of 1676), better than our prior expectation of 75%. Consent to take part in the telephone contact study was provided by 56% (946 of 1676, 95% CI = 54–59%) of participants, better than our prior assumption of 50%.

### Re‐randomizations

Of the 129 participants randomized more than once, 61 had received naloxone on the first occasion while 68 received control, consistent with expectation (64.5 each) if prior allocation did not influence the decision to be re‐randomized.

### Early cessation of randomization: decisions by the Trial Steering‐Data Monitoring Committee (TS‐DMC)

**Figure 3:**
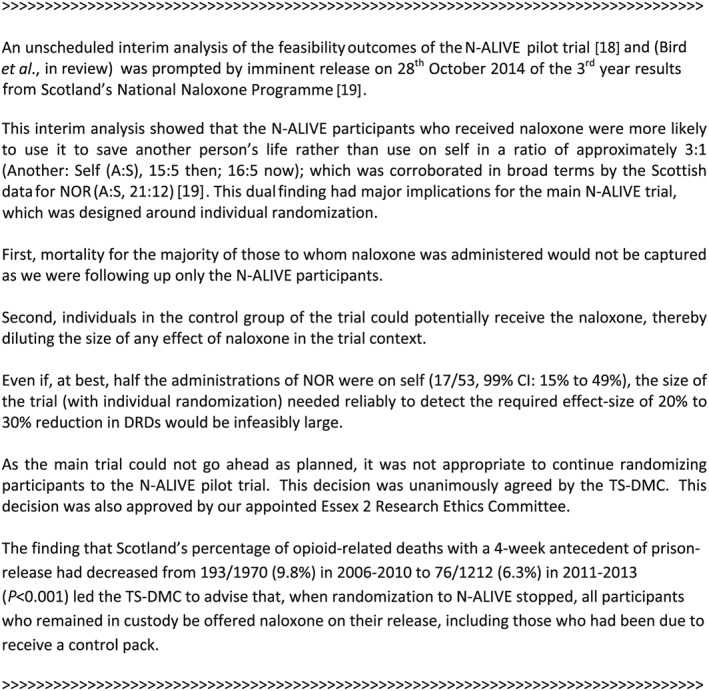
Explanation of the decision by the NALoxone InVEstigation (N‐ALIVE) Trial Steering—Data Monitoring Committee (TS‐DMC) to cease randomization in the N‐ALIVE pilot trial

We closed the trial to accrual on 8 December 2014, ahead of our planned closure date (Bird *et al*. in review). An unscheduled interim analysis of the feasibility outcomes of the N‐ALIVE pilot trial was prompted by the release on 28 October 2014 of the third year of results from Scotland's National Naloxone Programme 19; see Fig. 3.

When randomization to N‐ALIVE stopped, all participants who remained in custody were to be offered naloxone on their release, including those due to receive a control pack. The Principal Investigators at our prisons so agreed. Participants were otherwise followed‐up as per protocol.

### Baseline characteristics

The baseline characteristics and treatment allocation of the 1557 randomized participants who were released by 8 December 2014 (93% of the 1676 who were eligible to receive a pack on release) are shown in Table 2.

### Provision of packs on release

Of those participants released before 8 December 2014, 81% received their N‐ALIVE pack on release (1266 of 1557; 95% CI = 79–83%).

### RPSQs: return‐rate and findings

We received 218 of 1557 (14%) RPSQs by 8 December 2014, 205 with information on treatment assignment (112 naloxone; 93 controls), consistent with our prior expectation of 207 (95% CI = 179–235); see Table 1. Median and mean time [standard deviation (SD)] from release date to RPSQ completion were 64 and 80 (62) days.

- *Naloxone carriage and administration*

Of RPSQ respondents assigned to NOR, 76% (85 of 112; 95% CI = 68–84%) told a family member or friend about their naloxone (Table 3) and 71% (80 of 112; 95% CI = 63–79%) reported carriage of naloxone in the first 2 weeks post‐release, consistent with prior expectation (Table 1). Naloxone acquisition from other sources occurred at a non‐differential low rate (12 of 205, 95% CI = 2.6–9.1%). More often, RPSQ respondents reported that their NOR had been administered to ‘save’ someone else (14%; 16 of 112) than themselves (5%; five of 112); see also Fig. 3.

**Table 3:** Responses to returned prisoner self‐questionnaire.

Self‐questionnaire	NOR	Control	Total
Number of forms completed	112 (51%)	93 (43%)	205
Time from previous release to completion of questionnaire (days)	Mean 79, SD 59	Mean 85, SD 66	Mean 82, SD 63
	Median 64	Median 64	Median 64
	IQR = 37–108	IQR = 39–119	IQR = 38–108
Told family member/friend about naloxone	76% (85/112)	NA	NA
Told someone about naloxone	79% (89/112)	NA	NA
Carriage rate of naloxone	71% (80/112)	NA	NA
How often did you carry it?			
All	76% (61/80)		
Most	16% (13/80)	NA	NA
Some	6% (5/80)		
No response	1% (1/80)		
What did you do with the naloxone?		NA	NA
Saved other	14% (16/112)		
Saved self	5% (5/112)		
Lost it	13% (14/112)		
Had it taken away/stolen	10% (11/112)		
Given it away	7% (8/112)		
Thrown it away	2% (2/112)		
Broke the syringe	2% (2/112)		
No answer given	48% (54/112)		
Heroin use in the first 2 weeks after leaving prison			
(yes or no)	69% (77/112)	65% (60/93)	67% (137/205)
(Smoke/inject)	66% (74/112)	65% (60/93)	65% (134/205)
	*n* = 74	*n* = 60	*n* = 134
	Mean 9/14 days,	Mean 8/14 days,	Mean 9/14 days,
	SD 5 days	SD 5 days	SD 5 days
(Inject)	58% (65/112)	43% (40/93)	51% (105/205)

	*n* = 65	*n* = 40	*n* = 105
	Mean 9/14 days,	Mean 10/14 days,	Mean 9/14 days,
	SD 5 days	SD 5 days	SD 5 days
(Inject, alone)	23% (26/112)	29% (27/93)	26% (53/205)

	*n* = 25	*n* = 26	*n* = 51
	Mean 6/14 days,	Mean 6/14 days,	Mean 6/14 days,
	SD 5 days	SD 4 days	SD 4 days
Self‐overdose <2 weeks of release			
Overdose*	7% (8/112)	2% (2/93)	5% (10/205)
Someone present	8/8	1/2	9/10
Naloxone given	3/8	2/2	5/10
Taken to hospital	4/8	2/2	6/10
Self‐overdose >2 weeks of release			
Overdose	4% (5/112)	6% (6/93)	5% (11/205)
Someone present	3/5	6/6	9/11
Naloxone given	3/5	3/6	6/11
Taken to hospital	4/5	4/6	8/11
Presence at overdose of others <2 weeks of release			
Present	17% (19/112)	13% (12/93)	15% (31/205)
Naloxone given	10/19	1/12	11/31
Taken to hospital	12/19	8/12	20/31
Survived	17/19	11/12	28/31
Presence at overdose of others >2 weeks of release			
Present	15% (17/112)	15% (13/93)	15% (30/205)
Naloxone given	7/17	2/13	9/30
Taken to hospital	9/17	12/13	21/30
Survived	13/17	11/13	24/30
Naloxone acquisition‐rate χ^2^ on 1 d.f. = 1.46 *P* = 0.226	4% (5/112)	9% (8/93)	6% (13/205)
Do you think taking part in N‐ALIVE changed your own use of heroin in the first 2 weeks after release?			
No	38% (43/112)	65% (60/93)	50% (103/205)
Safer heroin use	54% (60/112)	32% (30/93)	44% (90/205)
Riskier heroin use	2% (2/112)	3% (3/93)	2% (5/205)

* Fisher's exact test: *P* = 0.116. NOR = naloxone‐on‐release; SD =standard deviation; NA = not applicable; N‐ALIVE = NALoxone InVEstigation.

Twenty‐one per cent (23 of 112) reported administration of naloxone to themselves or another before the arrival of a doctor or ambulance versus 9% (eight of 93) for controls (χ^2^ on 1 d.f. = 5.64; *P* = 0.02). Cumulative accounting for overdose victims being taken to hospital was 26% (29 of 112) for NOR versus 28% (26 of 93) for controls; χ^2^ on 1 d.f. = 0.11 (*P* = 0.74).

*Personal drug use, injecting and overdose events*

Two‐thirds of RPSQ respondents had used heroin in the first fortnight post‐release (67%, 137 of 205).

Half (105 of 205) of the RPSQ respondents had injected in the first fortnight—58% (65 of 112) for NOR and 43% (40 of 93) of controls; 26% (53 of 205; 95% CI = 20–32%) had injected when alone during the first 2 weeks post‐release, on a mean of 6 days out of 14 (95% CI = 4.9–7.1 days), broadly consistent with our prior expectation of someone else present at 80% of opiate overdoses (Table 3; Table 1).

Five per cent of RPSQ respondents (10 of 205) had personally experienced an overdose within the first fortnight—7% (eight of 112) for NOR and 2% (two of 93) of controls (Fisher's exact test, *P* = 0.12). Thereafter, until re‐imprisonment, the proportion personally overdosing was 5% (11 of 205) for NOR and controls alike.

*Witnessed overdoses and actions taken*

Fifteen per cent of RPSQ respondents (31 of 205) had personally witnessed an overdose during the first fortnight post‐release—17% (19 of 112) for NOR and 13% (12 of 93) of controls. Thereafter, until re‐imprisonment, the witness proportion was 15% (30 of 205) for NOR and controls alike.

*Opinions about NOR and the N‐ALIVE trial*

Taking part in N‐ALIVE was associated positively with safer heroin use by 60 of 112 (54%) RPSQ respondents randomized to NOR and 30 of 93 (32%) of controls (χ^2^ on 1 d.f. = 9.37, *P* < 0.002). Suggestions made by 113 RPSQ respondents were most commonly: ‘everyone should get naloxone’ (22); ‘everyone should get naloxone not just 50 : 50’ (10); ‘availability and access’ (11); ‘safer perception’ (8); ‘education and awareness’ (8); and ‘research trial a good idea’ (6); see Supporting information. Fifty difficulties were cited, the top three being: ‘didn't get naloxone’ (12), ‘no pack on release’ (7) and ‘police not aware’ (6).

Four difficulties were potential adverse events: (1) because police were unaware of the N‐ALIVE pilot trial, one ex‐prisoner would have been arrested but for intercession by a drug intervention programme worker; (2) ex‐prisoner's partner was worried about children finding his naloxone; (3) acquaintance of another ex‐prisoner took the naloxone ‘to see if he got a buzz from it’; and (4) ex‐prisoner was unsure how much of naloxone to administer to a person who had overdosed.

### Risk score

Table 4 shows that RPSQ respondents’ mean risk score was not significantly different between NOR (3.9) and controls (3.5). We observed an interaction (*P* = 0.049) between randomized assignment, self‐reported safer behaviour and mean risk score: for controls, but not for NOR, RPSQ respondents’ mean risk score was significantly lower for those who self‐reported safer behaviour (2.4) versus not (4.0).

**Table 4:** Risk score comparison, based on answers to returned prisoner self‐questionnaire.

Risk score comparison (lower score, less risky)	*n*	Median	Mean	SD	SE (difference)	Observed difference (95 % CI for difference)
Random assignment						
NOR	112	4.00	3.86	3.34	0.48	0.34
Control	93	2.00	3.52	3.43		(−0.59 to 1.27)
Safer behaviour as N‐ALIVE participant?						
No change/unsafe	115	3.00	3.87	3.54	0.47	0.38
Safer	90	4.00	3.49	3.18		(−0.54 to 1.30)
Safer behaviour as N‐ALIVE participant? (answers by those assigned to control group)						
No change/unsafe	63	3.00	4.03	3.50	0.71	1.60*
Safer	30	2.00	2.43	3.07		(0.20 to 3.00)
Safer behaviour as N‐ALIVE participant? (answers by those assigned to NOR group)						
No change/unsafe	52	3.50	3.67	3.61	0.64	–0.35*
Safer	60	4.00	4.02	3.12		(−1.61 to 0.91)

Test for interaction: difference in differences* [control– naloxone‐on‐release (NOR)] = 1.94. Standard error (SE) for difference in differences* (control‐NOR) = 0.98. Hence, 95% confidence interval (CI) for difference in differences* is from 0.008 to 3.876 (*P =* 0.049). SD= standard deviation; N‐ALIVE = NALoxone InVEstigation.

### Drug‐related deaths

Only half the DRDs registered in England and Wales in a specific calendar year actually occur during that calendar year 20, 21 and so it was necessary to wait at least a year to report on DRDs.

Nine deaths had occurred in the 12 weeks post‐release among 1557 randomized participants and were registered with the Office for National Statistics before 21 April 2016 (Table 1 and Supporting information). Five were DRDs, consistent with our null expectation of 9.7 DRDs in 12 weeks after release; but only two DRDs occurred in the first 4 weeks, well below our null expectation of 7.9 (Table 1).

Of the four opioid‐related DRDs, three were randomized to NOR, one of whom was released without his pack. The participant whose DRD was not opioid‐related was also randomized to NOR and had been released without his pack.

## Discussion

The N‐ALIVE pilot trial has randomized more prisoners than any other prison‐based, individually randomized controlled trial in Europe. We have shown that large‐scale trials of public health interventions are feasible within prisons. Prisoners themselves showed enthusiasm for the N‐ALIVE trial—their consent rate was excellent (72%).

The N‐ALIVE pilot trial stopped early because its own data, together with those from Scotland's National Naloxone Programme, were persuasive that approximately two‐thirds of NOR administrations were not to the ex‐prisoner for whom NOR was assigned. We had no means of knowing the identities of these other people: confounding of N‐ALIVE's control group could have occurred. The N‐ALIVE pilot trial ceased because individualized randomization to NOR cannot offer a clear‐cut answer: other trial designs are required.

We were concerned that a 50% consent rate combined with a 50% contact rate for the half randomized to actual telephone contact would mean that only 195 telephone interviews would be likely to be achieved from 1557 randomized participants. In practice, the achievement was lower still, with 81 successful telephone interviews. By contrast, RPSQs which were designed specifically to protect the respondent's confidentiality achieved their anticipated response rate.

Other prior assumptions were vindicated by the feasibility trial. Few actual or potential adverse events were reported: one reply card informed us that naloxone had been administered to an overdose victim who had survived but had experienced withdrawal symptoms; one ex‐prisoner had faced arrest because the police were not sufficiently aware of the N‐ALIVE trial; and two respondents cited their or a partner's concern for safer packaging lest children might access the naloxone.

Notwithstanding RPSQ respondents’ eight reports of overdose during the first fortnight for NOR versus two for controls, and comparable injection rates during the first fortnight post‐release (Table 3), RPSQ respondents randomized to NOR self‐reported safer heroin use compared with controls. However, our risk score comparisons (Table 4) showed a significant interaction whereby only for controls did the mean risk score align with self‐reported safer behaviour. Returned prisoners randomized to NOR perceived greater safety than their RPSQ answers demonstrated, which suggests some risk compensation about which Strang *et al*. 22 forewarned; see also other prevention policies, from seat‐belt legislation to safety helmets 23, 24, 25, where a degree of risk compensation detracted in a small way from the policy's overall benefit. N‐ALIVE participants’ main suggestion was that naloxone should be made available more widely to all those at risk.

To our knowledge, no previous contemporaneous before/after policy evaluation and randomized trial of effectiveness has had the same primary outcome: here, DRDs or opioid‐related DRDs with a 4‐week antecedent of prison release 6, 9, 14. The N‐ALIVE team convened its TS‐DMC ahead of the release of the third‐year results from Scotland's National Naloxone Programme 6, 19. Consistency between RPSQ responses on the administration of NOR (another versus self, 15 : 5) and Scotland's data on the utilization of NOR by those who applied for re‐supply (21 : 12) convinced the TS‐DMC that an individually randomized main trial was infeasible because only one‐third of NOR administrations was to the ex‐prisoner and two‐thirds to another person whose identity was unknown to the N‐ALIVE trial.

No reliable inference about NOR's effectiveness for reducing DRDs in the 12 weeks post‐release can be drawn from the early‐cessation N‐ALIVE pilot trial: five registered DRDs (four randomized to NOR, two of whom were released without their pack) were fewer than our a priori null expectation (9.7), perhaps because the expectation was too high rather than as a reflection of NOR's effectiveness. However, the 15% fatality rate (95% CI = 6–24%) at overdoses which our RPSQ respondents witnessed, typically within 12 weeks of their N‐ALIVE release date, suggests that our ex‐prisoners were present at higher‐fatality risk overdoses than suggested by an evidence synthesis, which gave a 6% fatality rate (95% CI = 2–11%) at witnessed opioid overdoses 6. Explanations for the higher fatality rate reported by our recidivists range from chance through assortative mixing of ex‐prisoners—who then share the same high DRD rate post‐release—to ex‐prisoners’ high DRD risk being due to a higher fatality rate per overdose (rather than to higher overdose rate with common fatality rate per overdose).

The Scottish results 14, 15, data on the cost‐effectiveness of naloxone 14, 26 WHO recommendations on naloxone 8, the United Kingdom's legal change on provision of naloxone 27, England's increase in opioid‐related DRDs 28 and prisoners’ support for initiatives such as the N‐ALIVE pilot trial make it timely for England and others to introduce a funded national naloxone policy; but also to evaluate, as did Scotland. In summer 2016, the National Institute for Health Research issued an evaluation call for naloxone studies in England. This call could address alternative, licensed non‐injectable formulations of naloxone or divert attention from England's failure to fund a naloxone policy: NOR with, or without, take‐home naloxone.

Our findings add trial‐based evidence to the growing consensus 15, 27 that pre‐provision of take‐home emergency naloxone can enable life‐saving interim measures to prevent overdose deaths, and that the period after prison release is not only a time of great concentration of such deaths but also of opportunity to prevent this major contribution to the global burden of disease 29.

### Research in context

#### Evidence before this study

The N‐ALIVE pilot trial was the first randomized controlled trial to investigate provision of naloxone‐on‐release (NOR) to prisoners who have previously injected heroin. The MRC funded the N‐ALIVE pilot trial to investigate the feasibility of a fully powered randomized trial.

#### Added value of this study

The N‐ALIVE pilot trial has shown that it is feasible to conduct a large prison‐based randomized controlled trial, with good participation from both prisons and prisoners. The finding that two‐thirds of administrations of NOR were to someone unknown to the N‐ALIVE pilot trial means that alternative research designs should be considered for preventative interventions against fatal overdose.

#### Implications of all the available evidence

The N‐ALIVE pilot trial has demonstrated the feasibility of recruiting many prisons, and consenting large numbers of prisoners, to take part in randomized evaluations that matter to prisoners. The practicality of NOR has now been demonstrated in two prison systems (by Scotland's National Naloxone Policy and by the N‐ALIVE pilot trial) and so prisons, internationally, can deliver NOR.

Scotland's non‐randomized before/after policy evaluation demonstrated the effectiveness of NOR and community‐based take‐home naloxone (jointly) for reducing opioid‐related deaths during the 4 weeks after prison release. Two‐thirds of administrations of NOR (Scotland; N‐ALIVE) were to someone other than the ex‐prisoner for whom NOR was prescribed. The N‐ALIVE pilot trial reacted promptly to external data from Scotland and from the trial itself to close the N‐ALIVE trial to recruitment.

Between 6000 and 8000 drug‐induced deaths are reported in Europe every year, with opioids their major cause. The European Monitoring Centre for Drugs and Drug Addiction exhorts Member States that many of these deaths could be prevented by adequate peer intervention using naloxone. Age‐related rising numbers of opioid‐related DRDs complicate before‐and‐after evaluations, as in Scotland.

### Clinical Trial Registration

N‐ALIVE pilot TRIAL: ISRCTN34044390.

### Declaration of interests

M.K.B.P., L.C., A.M.M.: none. J.S. is a researcher and clinician who has worked with a range of types of treatment and rehabilitation service‐providers, including treatments within prison and on prison release. J.S. is supported by the National Institute for Health Research (NIHR) Biomedical Research Centre for Mental Health at South London and Maudsley NHS Foundation Trust and King's College London. He has also worked with a range of governmental and non‐governmental organizations, and with pharmaceutical companies to seek to identify new or improved treatments (including naloxone products) from whom he and his employer (King's College London) have received honoraria, travel costs and/or consultancy payments. This includes work with, during the past 3 years, Martindale, Reckitt‐Benckiser/Indivior, MundiPharma, Braeburn/MedPace and trial medication supply from iGen. His employer (King's College London) has registered intellectual property on a novel buccal naloxone formulation with which J.S. is involved. J.S. has also been named in a patent registration by a Pharma company as inventor of a concentrated nasal naloxone spray. For a fuller account, see J.S.'s web‐page at: http://www.kcl.ac.uk/ioppn/depts/addictions/people/hod.aspx. S.M.B. served on Scotland's National Naloxone Advisory Group and co‐authored the peer‐review paper on before/after evaluation at 3 years of Scotland's National Naloxone Policy. S.M.B. holds GlaxoSmithKline shares.

## Supporting information


**Figure S1** NALoxone InVEstigation (N‐ALIVE) pack.
**Figure S2** Consolidated Standards Of Reporting Trials (CONSORT) Diagram and map of NALoxone InVEstigation (N‐ALIVE) prisons.
**Figure S3 (cont.)** Map of NALoxone InVEstigation (N‐ALIVE) prisons and the mental health research network hubs.
**Figure S3** Waiting‐time distribution from randomization date to release date with administrative censoring applied at 19 June 2015 (participants randomized and released before 8 December 2014).
**Table S1** Treatment allocation on re‐randomization.
**Table S2** Baseline characteristics for 1676 participants randomized and not withdrawn.
**Table S3** Summary of participants randomized at each participating prison.
**Table S4** Components of the NALoxone InVEstigation (N‐ALIVE) risk score using returned prisoner self‐questionnaires (RPSQ) questions; and risk score comparison.
**Table S5:** Nine registered deaths in the NALoxone InVEstigation (N‐ALIVE) pilot trial in the 12 weeks after release: based on deaths registered with the Office for National Statistics by 19 April 2016.
**Table S6** Summary of improvements suggested by participants who completed returned prisoner self‐questionnaires (RPSQ).
**Table S7** Summary of difficulties raised by participants who completed returned prisoner self‐questionnaires (RPSQ).
**Table S8** Responses to telephone questionnaire.

Data 1 Supporting info item
