# Supplementary material for: Randomized controlled pilot trial of naloxone‐on‐release to prevent post‐prison opioid overdose deaths
Source: Addiction. 2016 Dec 13;112(3):502–15. doi: 10.1111/add.13668 (PMC5324705; doi:10.1111/add.13668)
Supplement: Supplementary file 1 — Data 1 Supporting info item [file ADD-112-502-s001.doc]

**Supplementary Material**

**Figure S1: N-ALIVE pack**

**
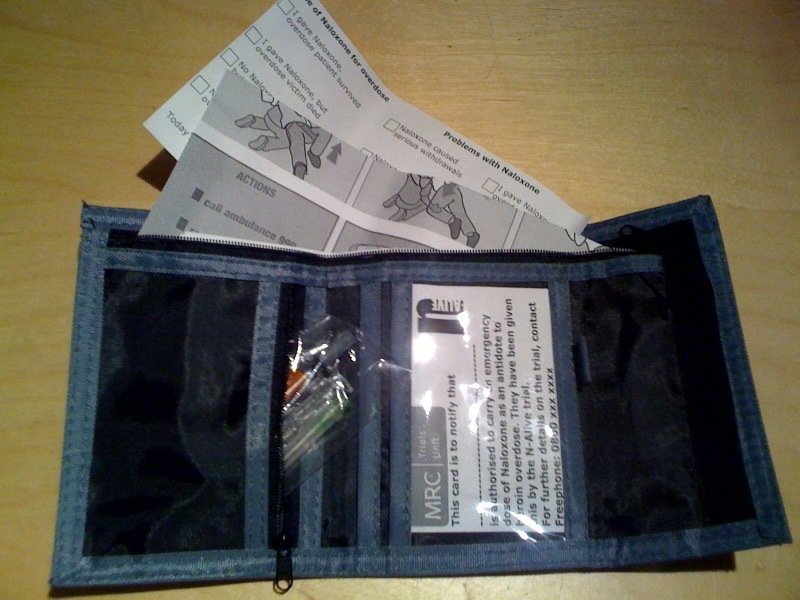
**

The wallet in the control pack did not contain a syringe but had an information leaflet describing the dangers of heroin overdose, advice on overdose management, a description of how to use 0.8mg of the 2.0mg naloxone and of safe syringe disposal, a card giving the N-ALIVE website details, a colour-coded pre-paid reply card and a pen.

**Figure S2: CONSORT Diagram and Map of N-ALIVE prisons**


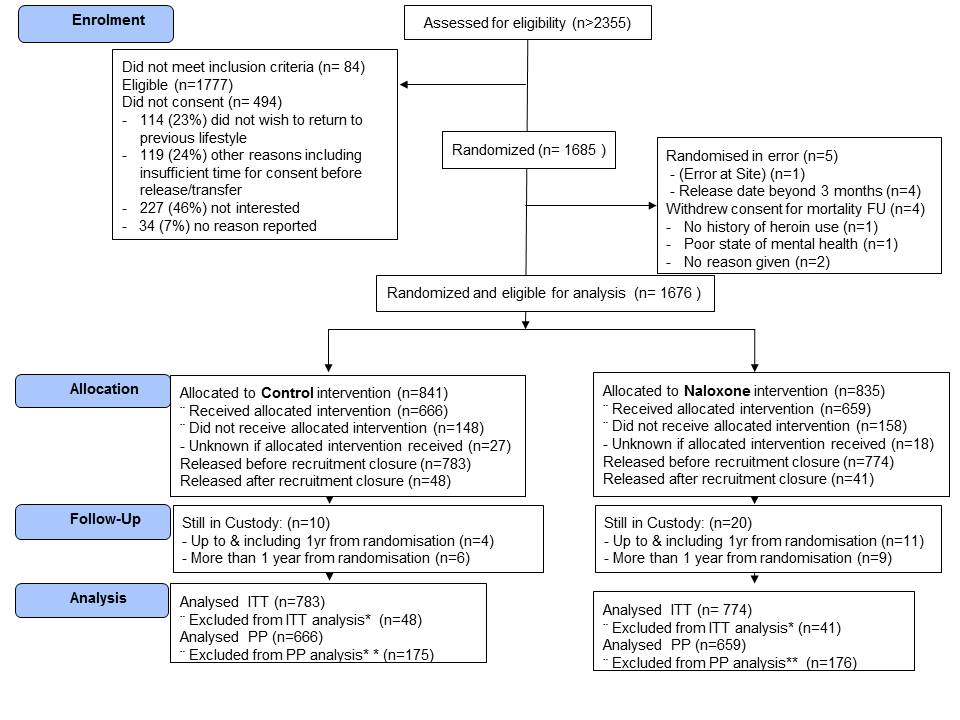


NB. Screening records have only been kept since Sep 2012 so only provide a snapshot of the proportions deemed eligible and subsequently randomized.

* Excluded from ITT analysis participants released after recruitment closure (n=48, 40)

** Included in PP analysis participants released with pack only

**Figure 3 (cont): Map of N-ALIVE Prisons and the Mental Health Research Network Hubs**


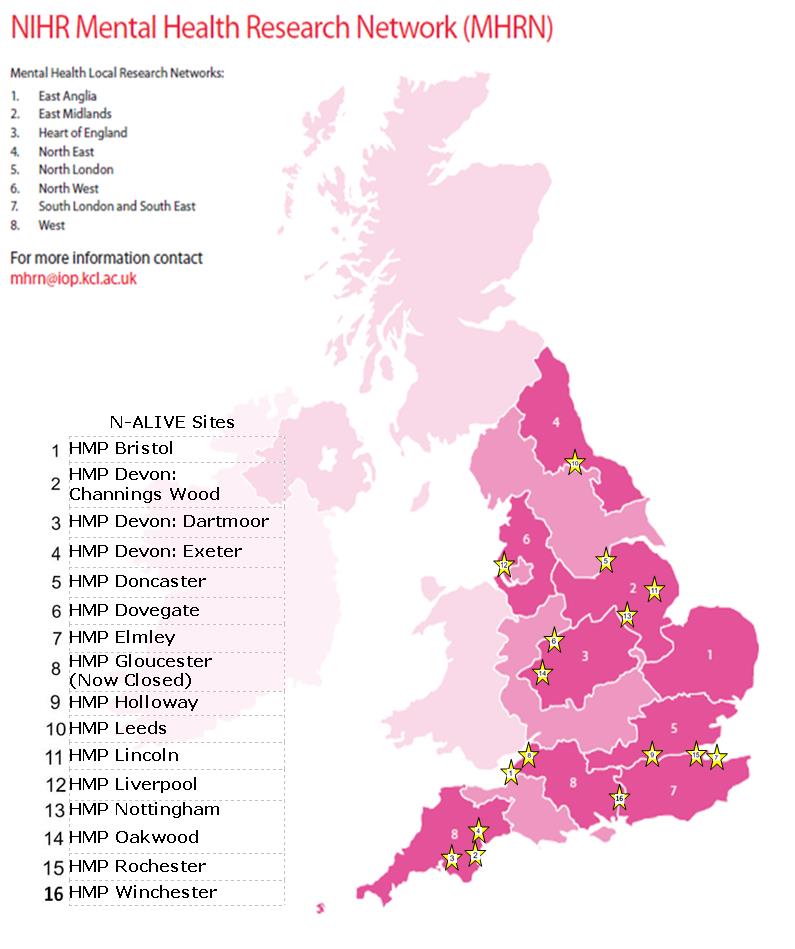


**Releases and Provision of Packs on Release**

Of those participants released before 8^th^ December 2014, 81% received their N-ALIVE pack on release (1266/1557; 95% CI: 79% to 83%).

Consistent with their eligibility, 91% of participants were released within three months of randomization-date and 95% within six months [**Figure S3**]; 50^th^, 75^th^ and 90^th^ percentiles for time-to-release were 14, 39 and 79 days.

**Figure S3: Waiting-time distribution from randomization-date to release-date with administrative censoring applied at 19 June 2015 (Participants Randomized &Released before Dec 8, 2014)**

**Table S1: Treatment Allocation on Re-randomization**

| Patients randomized twice | Treatment Sequence | Number |
| --- | --- | --- |
| 120 | CN | 38 |
|  | CC | 28 |
|  | NC | 38 |
|  | NN | 16 |
| Patients randomized three times | Treatment Sequence | Number |
| 8 | CNN | 2 |
|  | NCN | 2 |
|  | NNC | 2 |
|  | NNN | 2 |
| Patient randomized four times | Treatment Sequence | Number |
| 1 | NCNC | 1 |

**C = control, N = naloxone-on-release**

**Table S2: Baseline Characteristics for 1676 Participants Randomized and Not Withdrawn**

|  | **NOR** | | **Control** | | **All** | |
| --- | --- | --- | --- | --- | --- | --- |
| **Age (Mean & s.d.) years** | Mean 35 years  s.d. 7 years | | Mean 35 years  s.d. 6 years | | Mean 35 years  s.d. 7 years | |
|  | N | % | N | % | N | % |
| **Age categories (N, %) years**  **18-24**  **25-34**  **35-44**  **45+** | 42  414  319  49 | 5  50  39  6 | 41  419  321  51 | 5  50  39  6 | 83  833  640  100 | 5  50  39  6 |
| **Gender (N, %)**  **Males**  **Females** | 822  13 | 98  2 | 826  15 | 98  2 | 1648  28 | 98  2 |
| **Treatment for Addiction (at Randomization) (N, %)**  **Opiate substitution**  **Opiate detoxification**  **Other (eg Naltrexone, no current treatment)**  **Not recorded** | 534  176  124  1 | 64  21  15  <1 | 542  175  123  1 | 64  21  15  <1 | 1076  351  247  2 | 64  21  15  1 |
| **Likely Incarceration Interval (N,%)**  **(Date of randomization-Expected Release Date at randomization)**  **Within 28 days**  **4-12 weeks**  **>12weeks**  **Unknown release date** | 558  187  20  70 | 67  22  2  8 | 562  194  16  69 | 67  23  2  8 | 1120  381  36  139 | 67  23  2  8 |

**Table S3: Summary of Participants Randomized at each Participating Prison**

| **Prison** | **Participants Randomized**  **(n)** | **Participants Randomized and not withdrawn**  **(n)** | **Participants Randomized and Released by Dec 8th, 2014**  **(n)** | **Participants**  **Randomized and Released with Pack by Dec 8, 2014**  **(n)** | **Months open to recruitment (to end of Nov 2014)** |
| --- | --- | --- | --- | --- | --- |
| HMP Bristol* | 368 | 366 | 327 | 224 | 25 |
| HMP Channing’s Wood | 40 | 40 | 40 | 31 | 26 |
| HMP Dartmoor | 18 | 18 | 18 | 15 | 26 |
| HMP Doncaster | 84 | 84 | 83 | 79 | 17 |
| HMP Dovegate* | 161 | 159 | 132 | 101 | 25 |
| HMP Elmley | 1 | 0 | 0 | 0 | 0 |
| HMP Exeter | 110 | 110 | 103 | 89 | 26 |
| HMP Gloucester | 11 | 11 | 10 | 10 | 5 |
| HMP Holloway | 28 | 28 | 24 | 24 | 11 |
| HMP Leeds | 126 | 124 | 116 | 109 | 11 |
| HMP Lincoln* | 99 | 99 | 92 | 78 | 26 |
| HMP Liverpool | 228 | 226 | 219 | 180 | 19 |
| HMP Nottingham | 288 | 288 | 274 | 268 | 31 |
| HMP Oakwood | 4 | 4 | 4 | 1 | 10 |
| HMP Winchester* | 119 | 119 | 115 | 57 | 26 |
| **Total** | **1685** | **1676** | **1557** | **1266** | **284** |

*Prisons where remand prisoners were randomized

**Table S4: Components of N-ALIVE Risk Score using RPSQ questions; and Risk Score Comparison**

| **Questions on Heroin Use & Assigned Risk Score** | | | | |
| --- | --- | --- | --- | --- |
|  |  | **No response/No** | **Yes** |  |
| **In the first 2 weeks after leaving prison, did you…** | **Smoke or Inject Heroin** | 0 | 1 |  |
|  | **Inject Heroin** | 0 | 1 |  |
|  | **Inject Heroin alone** | 0 | 1 |  |
| **If yes, on how many days out of 14?** | **Number of Days Smoke or Inject Heroin** | 0 (No response/NA/0 days) | 1 (1-6 days) | 2 (7-14 days) |
|  | **Number of Days Inject Heroin** | 0 (No response/NA/0 days) | 1 (1-6 days) | 2 (7-14 days) |
|  | **Number of Days Inject Heroin alone** | 0 (No response/NA/0 days) | 1 (1-3 days) | 2 (4-14 days) |
| **Questions on Overdose & Assigned Risk Score** | | | | |
|  |  | **No response/No** | **Yes** |  |
| **Self-Overdose <=2 weeks of release** | Overdose | 0 | 2 |  |
|  | Someone present | 0 | -1 |  |
|  | Naloxone given | 0 | -1 |  |
| **Self-Overdose**  **>2 weeks of release** | Overdose | 0 | 2 |  |
|  | Someone present | 0 | -1 |  |
|  | Naloxone given | 0 | -1 |  |
| **Questions on Witnessing Overdose & Assigned Risk Score** | | | | |
|  |  | **No response/No** | **Yes** |  |
| <=2 weeks of release | Present | 0 | 1 |  |
|  | Naloxone given | 0 | -1 |  |
|  | Survived overdose | 0 | -1 |  |
| >2 weeks of release | Present | 0 | 1 |  |
|  | Naloxone given | 0 | -1 |  |
|  | Survived overdose | 0 | -1 |  |
| **Question on Naloxone Acquisition** | | | | |
|  |  | **No response/No** | **Yes** |  |
|  | Naloxone Acquisition | 0 | -1 |  |

| **Risk Score Comparison**  **(lower score, less risky)** | **N** | **median** | **Mean** | **sd** | **Se(Diff)** | **Observed Difference**  **(95% CI for Difference)** |
| --- | --- | --- | --- | --- | --- | --- |
| **Random Assignment** | | | | | | |
| NOR | 112 | 4.00 | 3.86 | 3.34 | 0.48 | 0.34 |
| Control | 93 | 2.00 | 3.52 | 3.43 |  | (-0.59 to 1.27) |
| **Safer behaviour as N-ALIVE participant?** | | | | | | |
| No change/Unsafe | 115 | 3.00 | 3.87 | 3.54 | 0.47 | 0.38 |
| Safer | 90 | 4.00 | 3.49 | 3.18 |  | (-0.54 to 1.30) |
| **Safer behaviour as N-ALIVE participant? (answers by those assigned to control group)** | | | | | | |
| No change/Unsafe | 63 | 3.00 | 4.03 | 3.50 | 0.71 | 1.60* |
| Safer | 30 | 2.00 | 2.43 | 3.07 |  | (0.20 to 3.00) |
| **Safer behaviour as N-ALIVE participant? (answers by those assigned to NOR group)** | | | | | | |
| No change/Unsafe | 52 | 3.50 | 3.67 | 3.61 | 0.64 | -0.35* |
| Safer | 60 | 4.00 | 4.02 | 3.12 |  | (-1.61 to 0.91) |
| **Test for Interaction:** Difference in differences* (Control-NOR)=1.94,  Se for Difference in differences* (Control-NOR) = 0.98 and so 95% CI from 0.008 to 3.876 (p=0.049) | | | | | | |

**Table S5: Nine registered-deaths in N-ALIVE pilot trial in the 12 weeks after release:** based on deaths registered with the Office for National Statistics by 19th April 2016.

| Age at Death (years) | Days from Release to Death  (release-day included) | **Cause of Death:**  **Underlying (ORD; DRD; non-DRD)**  All other mentioned causes  Substances mentioned in coroner’s text  **ORD = opioid-related DRD** | | Days from Death to Registration of Death  (death-day included; **R** if registered by 31 October 2014) | Randomly Assigned  (***** if released without N-ALIVE pack) |
| --- | --- | --- | --- | --- | --- |
| **Three registered-deaths occurred in the first 4 weeks post-release** **(2 ORDs)** | | | | | |
| 42 years | 2  days | **Underlying**  **(ORD)** | **X424 Accidental poisoning by and exposure to narcotics and psychodysleptics [hallucigens], not elsewhere classified, street and highway.** | 77  days | Naloxone |
|  |  | All other causes  Mentioned | T401 (Poisoning: heroin); T405 (Poisoning: cocaine) |  |  |
|  |  | Substances | Heroin and cocaine |  |  |
| 46 years | 11 days | **Underlying**  **(non-DRD)** | **U509 Accelerated death registration which is pending investigation by the police.** | 681  days | Control |
|  |  | All other causes  Mentioned | T71 (Asphyxiation)  X910 (Assault by hanging strangulation and suffocation, home) |  |  |
|  |  | Substances |  |  |  |
| 36 years | 15 days | **Underlying**  **(ORD)** | **X425** **Accidental poisoning by and exposure to narcotics and psychodysleptics [hallucigens], not elsewhere classified, trade and service area.** | 68**^R^**  days | Naloxone* (released without pack) |
|  |  | All other causes  Mentioned | T401 (Poisoning: heroin) |  |  |
|  |  | Substances | Heroin |  |  |
| **Six registered-deaths occurred during weeks 5-12 post-release (2 ORDs; 1 DRD)** | | | | | |
| 38 years | 29 days | **Underlying**  **(ORD)** | **X420** **Accidental poisoning by and exposure to narcotics and psychodysleptics [hallucigens], not elsewhere classified, home.** | 250  days | Naloxone |
|  |  | All other causes  Mentioned | T402 (Poisoning: other opioids),  F192 (Mental and behavioural disorders due to multiple drug use and use of other psychoactive substances, dependence syndrome) |  |  |
|  |  | Substances | Morphine |  |  |
| 44  Years | 42  days | **Underlying**  **(non-DRD)** | **K559 Vascular disorder of intestine, unspecified.** | 68**^R^**  Days | Naloxone |
|  |  | All other causes  Mentioned | J180 (Bronchopneumonia, unspecified)  K746 (Other and unspecified cirrhosis of liver)  K861 (Other chronic pancreatitis) |  |  |
|  |  | Substances |  |  |  |
| 33  Years | 50 days | **Underlying**  **(DRD)** | **X440 Accidental poisoning by and exposure to other and unspecified drugs, medicaments and biological substances, home.** | 174  days | Naloxone* (released without pack) |
|  |  | All other causes  Mentioned | T509 (Poisoning: other and unspecified drugs, medicaments and biological substances)  F191 (Mental and behavioural disorders due to multiple drug use and use of other psychoactive substances, harmful use) |  |  |
|  |  | Substances | Multiple drug toxicity |  |  |
| 37  Years | 61 days | **Underlying**  **(non-DRD)** | **I269 Pulmonary embolism without mention of acute cor pulmonale.** | 30  days | Control |
|  |  | All other causes  Mentioned | J969 (Respiratory failure, unspecified);  J849 (Interstitial pulmonary disease, unspecified) |  |  |
|  |  | Substances |  |  |  |
| 39  Years | 64  days | **Underlying**  **(non-DRD)** | **W130 Fall from, out of or through building or structure, home.** | 267  days | Naloxone |
|  |  | All other causes  Mentioned | T07 (Unspecified multiple injuries);  F111 (Mental and behavioural disorders due to use of opioids, harmful use);  F131 (Mental and behavioural disorders due to use of sedatives or hypnotics, harmful use);  R418 (Other and unspecified symptoms and signs involving cognitive functions and awareness). |  |  |
|  |  | Substances |  |  |  |
| 40  Years | 71  days | **Underlying**  **(ORD)** | **X420** **Accidental poisoning by and exposure to narcotics and psychodysleptics [hallucigens], not elsewhere classified, home.** | 295**^R^**  days | Control |
|  |  | All other causes  Mentioned | T403 (Poisoning: methadone); |  |  |
|  |  | Substances | Toxicity of prescribed medication (methadone) |  |  |
| **Furthermore, four registered-deaths have occurred at 85-183 days post-release**  **(1 ORD; 1 DRD), the wash-out period before permitted re-randomization** | | | | | |
| 30  Years | 112  days | **Underlying**  **(non-DRD)** | **J22 Unspecified acute lower respiratory infection** | 13**^R^**  days | Control |
|  |  | All other causes  Mentioned |  |  |  |
|  |  | Substances |  |  |  |
| 38  Years | 136 days | **Underlying**  **(non-DRD)** | **W800 Inhalation and ingestion of other objects causing obstruction of the respiratory tract, home.** | 95**^R^**  days | Control* (released without pack) |
|  |  | All other causes  Mentioned | J849 (Interstitial pulmonary disease, unspecified);  T179 (Foreign body in respiratory tract, part unspecified) |  |  |
|  |  | Substances |  |  |  |
| 28  Years | 180  days | **Underlying**  **(ORD)** | **X425** **Accidental poisoning by and exposure to narcotics and psychodysleptics [hallucigens], not elsewhere classified, trade and service area.** | 285  days | Naloxone*(released without pack) |
|  |  | All other causes  Mentioned | T403 (Poisoning: Methadone);  T401 (Poisoning: Heroin);  F112 (Mental and behavioural disorders due to use of opioids, dependence syndrome);  F102 (Mental and behavioural disorders due to use of alcohol, dependence syndrome). |  |  |
|  |  | Substances | Methadone and heroin |  |  |
| 39  Years | 115  days | **Underlying**  **(DRD)** | **F191 Mental and behavioural disorders due to multiple drug use and use of other psychoactive substances, harmful use.** | 203  days | (1^st^ release) Naloxone but not released with pack, (2^nd^ release) Control upon second release & released with pack.** |
|  |  | All other causes  Mentioned | G931, I639, I469, F101 (Mental and behavioural disorders due to psychoactive substance use, harmful use) |  |  |
|  |  | Substances | Died as a consequence of drug and alcohol abuse. |  |  |

**Timeline pertains to the participant’s 2^nd^ release when he was randomized to control.

**Table S6: Summary of Improvements suggested by Participants who completed RPSQ**

| **Question not answered** | **65** |
| --- | --- |
| **No suggestions for improvement** | **37** |
| **N/A** | **3** |
| **Everyone should get Naloxone** | **22** |
| all participants should be given the naloxone | 1 |
| Everyone needs a pack on release | 1 |
| Everyone should be allowed a Naloxone pack | 1 |
| Everyone should get the Naloxone | 12 |
| I just think every user should have one and everyone knows the risks | 1 |
| I think every user should have naloxone | 1 |
| None except that everyone should get it. | 1 |
| The more people that have it the better | 1 |
| They should give it to everyone thank you very much | 1 |
| To give the injection to everyone | 1 |
| I think Everybody should be released with the actual injection. Thank you | 1 |
| **Everyone should get Naloxone, not just 50:50** | **10** |
| Everybody should get given it rather than just 50:50 chance of getting it. | 1 |
| Everybody should get it rather than the 50:50 chance | 1 |
| Everybody should get the Naloxone rather than only half getting it | 1 |
| Everyone should get it not just 50% | 1 |
| Give everybody Naloxone not 50:50 chance | 1 |
| I think the 50/50 chance of receiving is cruel and / or dangerous | 1 |
| None other than everybody who takes part should get Naloxone rather than just half | 1 |
| That everybody gets given Naloxone rather than 50:50 | 1 |
| Thinks everybody should be given it on their release rather than 50:50 | 1 |
| thinks everybody should get it rather than 50:50 | 1 |
| **Everyone should get Naloxone - More Prisoners on Release** | **5** |
| Everybody should be given Naloxone on release from prison. | 1 |
| Give Naloxone as standard on discharge | 1 |
| Naloxone to be made available to everyone on release | 1 |
| should be given to every user upon release | 1 |
| The only improvement I can think of is to give it to more prisoners leaving jail. Thank you. | 1 |
| **Everyone should get Naloxone - history of overdose on release** | **4** |
| I think that all people who have been on heroin before coming to prison should be released with naloxone as a precaution as they are more likely to overdose than a user who uses daily | 1 |
| If people have a history of overdose on release then they should receive the pack the prison nurse's knew if previously od therefore should have been given the pack... | 1 |
| People who overdose in the past, or who are on heroin long - term should ALL have Naloxone | 1 |
| Thinks that Naloxone should be given to those most at risk of overdose (pervious history of over dosing) | 1 |
| **Everyone should get Naloxone - ALL, including non-users** | **2** |
| Give it to all inmates/every prison, it would make a difference | 1 |
| Give Naloxone to everyone including those who don't use drugs | 1 |
| **Everyone should get Naloxone, saved friend's life** | **3** |
| Give it to more people cause it saved my friends life | 1 |
| Give to as many heroin uses as it helped me save a life | 1 |
| I think that it's a good thing to give this to people when they leave prison. No, Thank You - you saved my mates life | 1 |
| **Everyone should get Naloxone, to help/save people** | **3** |
| Every heroin user leaving prison should be issued with the N-ALIVE pack as I do think it WILL save lives! Thank You! | 1 |
| Everyone should get a pack as I could of helped 2 people before paramedics came | 1 |
| Put naloxone in every pack because it would actually help people | 1 |
| **Everyone should get Naloxone + Safer Perception** | **2** |
| Everyone should get it, because it can be a deterrent from using and it can make you realise you could die. | 1 |
| Give it to all people who would need it or be around people who could use it! N-ALIVE did make me think to use around people I could trust because I did not have it in my wallet so it made me think safer | 1 |
| **Safer Perception** | **8** |
| Can't think of any but being given Naloxone should make you think more safer in using is it did me | 1 |
| Had I overdosed I had the safety net of Naloxone | 1 |
| It helped me to put down my gear knowing the N-ALIVE was always with me I fear overdose a lot more. | 1 |
| it made me and others around me feel a bit safer when injecting | 1 |
| Safer for people in my presents | 1 |
| Stayed clean and stayed away from users and dealers. Thanks | 1 |
| Stopped me from using IV | 1 |
| Would of been better and would of felt safer if I had one. | 1 |
| **Education & Awareness** | **8** |
| A lot more awareness of overdoses from heroin and other drugs | 1 |
| I hold group sessions to educate about naloxone and how it can save lives make naloxone more accessible | 1 |
| Make police more aware | 1 |
| Make police more aware of the trial | 1 |
| Training on how to administer naloxone properly | 1 |
| Training on how to use Naloxone | 1 |
| Try make the police more aware so it isn't taken away from people | 1 |
| Yes I think more people could be aware of what Naloxone is and that it is safe for trained people to carry and use it when needed. Drug users as well as non-drug users because after I was given this form a few of the lads from the wing asked and they were not sure what it was both drug and non-drug users. Thank you. | 1 |
| **Availability + Access** | **11** |
| I think it should be widely available for every heroin user as it does save lives and would save a lot more than just the life saved with it if it was available to everyone. | 1 |
| Improve people's lives - give it to more people. Make it available at the needle exchange. | 1 |
| It would be much better if Naloxone were more readily available or, if possible, have dedicated places where Naloxone could be obtained - as I witnessed an overdose personally, and know of 7 overdoses, 4 of which ended in fatalities. I believe all 4 people would have stood a chance of survival had Naloxone been available. Thank you for opening my eyes!! | 1 |
| more information to be able to get more naloxone after release | 1 |
| more people should get the naloxone treatment | 1 |
| more people should have access to it | 1 |
| Not really - thinks that needle exchanges should provide it incase people have to use the Naloxone or it expires | 1 |
| Should be made available for PPOs who are in and out of prison more | 1 |
| Target shared accommodation places or known drug places and give out Naloxone | 1 |
| To have naloxone available | 1 |
| Use of local pharmacy to replace if used | 1 |
| **Availability + Access & More Doses** | **1** |
| issue more Naloxone i.e. every pack - go to other sources to get Naloxone/replace it if used | 1 |
| **More doses of Naloxone** | **4** |
| As used the naloxone in first week of release would have liked more doses | 1 |
| Could have more naloxone | 1 |
| I think they should double up on the n-alive because sometimes it could be more than two people at once that go over. | 1 |
| putting the doses into separate syringes and give more syringes as if more people overdosed you wouldn't have to inject them with the same syringe - because there is enough in the pre-loaded syringe for more than one dose | 1 |
| **Carriage** | **3** |
| Attach Naloxone to a keyring so you can't leave it at someone else's house | 1 |
| have some sort of reminder to ensure I have in my possession 24/7 | 1 |
| To encourage people to carry it around maybe put in on a chain so can be carried around the neck - high chance of wallet being stolen | 1 |
| **Miscellaneous** | **3** |
| Job Centre need to help me more instead of just stopping my money | 1 |
| Suggested that participants could be followed up by N-ALIVE worker whilst they are on the out. | 1 |
| The first N-ALIVE I had I used the syringe for heroin. | 1 |
| **N-ALIVE Trial - Good Idea** | **6** |
| all good I think it's a good idea | 1 |
| Good idea that people have it on them | 1 |
| I think that the N-ALIVE program is a good idea | 1 |
| Nice wallet, useful information. Injections are a good thing for the right people | 1 |
| None - thought giving prisoners Naloxone on their release is a good thing | 1 |
| think it’s a good idea if you need it and if not at least it’s there | 1 |
| **N-ALIVE Trial: Randomized Allocation Disappointment** | **6** |
| because used heroin naloxone would have been useful | 1 |
| Everyone should get the drug (I didn’t) | 1 |
| Give the naloxone to users that genuinely want it | 1 |
| It wasn't in my pack and everyone else didn't have it. So it was useless really I did see someone o.d. in St Pauls park however ambulance had just arrived. I spoke to other people who were released as well as me just b4 + after. None of them got N-Alive at all. We all said it was pointless. | 1 |
| not really but disappointed didn't get the naloxone | 1 |
| Thinks heroin use would have been safer if had got the Naloxone | 1 |
| **N-ALIVE Trial - Syringe/Needle** | **3** |
| Better if the needle was fixed on the syringe | 1 |
| Safer packaging in case kids get to it | 1 |
| To place a plastic syringe instead of glass | 1 |
| **N-Alive Trial - 6 month re-randomization interval** | **2** |
| Be able to have it again no matter how long it was since the last time I was given it | 1 |
| no 6 month gap in between taking part again | 1 |
| **N-ALIVE Trial - Ensure Packs are given out on release** | **3** |
| Making sure participants have their wallets in their property ready for release. | 1 |
| come and give it out yourself and they can be no altercations | 1 |
| Make sure packs are given out upon release!!! | 1 |
| **N-ALIVE Trial - Other Suggestions** | **4** |
| A letter in the box saying that the wallet contained Naloxone (participant didn't check the pocket inside the wallet) | 1 |
| Background checks and give Naloxone to the riskier people | 1 |
| Get more people into the study - do a course on overdoses and what to do | 1 |
| Stated that the phone call could have gone into a bit more detail. | 1 |
| Grand Total | 218 |

**Table S7**: Summary of Difficulties Raised by Participants who completed RPSQ

| **Difficulties Raised – Summary Categories** | **Frequency** |
| --- | --- |
| **N/A** | **5** |
| **Question not answered** | **94** |
| **No difficulties** | **61** |
| **No difficulties, positive comments** | **8** |
| All very clear and useful. | 1 |
| I had no difficulties and I still have it safely put away to use if ever needed! | 1 |
| I had no difficulties it's a very helpful course | 1 |
| I had no difficulties or issues carrying Naloxone, it was a re-assurance. | 1 |
| I had no difficulties, as it was easy to carry & I would of used it if necessary | 1 |
| No difficulties very simple and helpful | 1 |
| No. But I think it is helpful and definitely could save lives. | 1 |
| No, no difficulties, understand it, I knew what it was and how to administrate it. | 1 |
| **Police/Community not aware of trial** | **8** |
| Lost it when got arrested - could possibly be because police weren't fully aware of the trial | 1 |
| Not many people know about Naloxone - had to keep explaining it to people | 1 |
| police told it away when I was arrested - had it for a week only | 1 |
| Police took it off him | 1 |
| Police weren't aware of study- tried to take it of me. In the end they did let me keep it. | 1 |
| The police took it away - didn't seem to be aware of the trial | 1 |
| The police weren't aware of the trial- almost got arrested but DIP worker intervened | 1 |
| The probation hostel would not allow me to have it and left it in my meds. This could have caused a death mine / others and I was disgusted by this Hostel | 1 |
| **Didn't get Naloxone** | **8** |
| **Didn't get Naloxone, overdosed or witnessed overdosed** | **4** |
| Didn't 'WIN' pack both myself and girlfriend overdosed twice I wish I had the pack as I could of done something | 1 |
| Not a difficulty but didn't get the Naloxone and overdosed, had to rely on ambulance to give Naloxone | 1 |
| 4 weeks after being released I witnessed an overdose but didn’t have the N-ALIVE kit, if I did I'd of given it to him | 1 |
| Bit upset that didn't get Naloxone as could have possibly saved somebody | 1 |
| **Everyone should get Naloxone on release** | **2** |
| Everyone who injects drugs should get Naloxone when released | 1 |
| Need to improve system so that everyone gets these packs on release. | 1 |
| **No pack on release** | **7** |
| **About N-ALIVE trial** | **2** |
| All 3 pieces weren't in the wallet - plunger was missing | 1 |
| Not being able to get it again because of the 6 month limit before you could get it again | 1 |
| **Carriage** | **2** |
| Don't usually carry a wallet so this is why didn't carry it when out and about | 1 |
| forgot to carry at all times | 1 |
| **Miscellaneous** | **17** |
| Didn’t get to use it and did not need to as I left it at brothers and didn’t overdose anyway | 1 |
| Have had no difficulties whilst out in the community | 1 |
| I didn't end up in any situations where its use was called for. | 1 |
| I didn't think I would use it because I smoked this time | 1 |
| I lost the needle I put it down the drain because I felt like using crack. | 1 |
| I made think about how risky it is to use and also think about my tolerance | 1 |
| I was staying at a mates house and one day when I was out someone who I know took the N-ALIVE to see if he got a buzz off it. I was out a week and did not use heroin | 1 |
| I was unsure to the amount to issue to the person having an overdose | 1 |
| I was weary about being stopped with it on my person. most importantly it made me decide NEVER to use again, (I haven't) | 1 |
| Job Centre stopping my money to live on that is why I am back in prison. | 1 |
| No difficulties, I think that everyone should do the Group | 1 |
| no support, no methadone on release | 1 |
| Not having naloxone available | 1 |
| Partner was achilles heal - made it more difficult to not use | 1 |
| Partner was worried about kids finding it | 1 |
| Prisoner - I add the naloxone in my property the reception so throw it in the bin not knowing or careing what it was | 1 |
| the demographics are too difficult to collect in this study due to the chaotic behaviour of the target group therefore the statistical value of the control group will not be present in the final hypothesis. Eliminate your control group and save more lives | 1 |
| **Grand Total** | **218** |

**METHODS**

**Telephone Contact Randomization**

At the outset we also asked participants to consent to a once-only phone-contact sub-study; those who consented were randomized further, in the ratio 2:1:1 between no contact and phone-contact in the 1^st^ versus 2^nd^ fortnight after release. The rationale for the 50% no-contact-rate was to minimize contamination of the main trial as the phone-interview asked participants if they were carrying naloxone - in effect, reminding them to do so.

**Pre-paid Reply Cards**

Via anonymous pre-paid reply-cards, we gave ex-prisoners or others present at an overdose the opportunity spontaneously to alert us to critical events, problems or risks as perceived by them. The reply cards were colour-coded – orange if the participant was randomized to naloxone, white if randomized to control.

**RESULTS**

**Recruitment: prisons and participants**

Consent for record linkage to establish the number of non-fatal overdose admissions (NFOAs) to accident and emergency departments in the 12 weeks after release was given by 1655 (99%) of the 1676 participants who were randomized and not withdrawn. Consent to complete the RPSQ was given by 85% of participants (1417/1676) (better than our prior expectation of 75%). Consent to take part in the phone-contact study was provided by 56% (946/1676; 95% CI: 54% to 59%) of participants (slightly better than our prior assumption of 50%).

**Telephone interviews in the 4-weeks post-release: consistency with RPSQs**

Eighty-one telephone interviews were carried out (36 naloxone, 45 controls; 43 in first fortnight, 38 in second fortnight), substantially below our prior expectation of 195 (see Table 6) despite up to five attempts to achieve contact in the assigned fortnight. The mean interval from release date to completion of phone questionnaires was 10 days (sd 3 days) for those randomized to contact in the first 2-weeks and 22 days (sd 3 days) for those randomized to the second 2-weeks.

There were no substantial delays in the MRC CTU team being informed about participants’ release-dates, which could have interfered with the timeliness of telephone contacts: the median delay was 1 day (interquartile range of 0 to 6 days) with 90^th^ percentile of 14 days, which coincides with the end of the first 2 weeks post-release.

Contemporaneous phone-contact interviews about heroin use in the past 3 days corroborated the information gathered on the RPSQ. Heroin use was reported by 38% (31/81) of telephone respondents – 47% (17/36) of those in the naloxone group and 31% (14/45) of those in the control group. Telephone interviews also found that, of the 31 who had used heroin, 10 (32%) had injected when alone - 6% (2/36) and 18% (8/45) for naloxone and control groups respectively. This was broadly consistent with our prior expectation that someone else is present at 80% of opiate overdoses. (See **Table S8** for a summary of findings from the telephone interviews).

**Reply Cards**

Four reply-cards were received by 8th December 2014 (versus nine expected from 1557 randomized and released): two gave notification that naloxone had been administered and the overdose victim had survived but one of these two survivors had experienced withdrawal symptoms; two were control reply-cards, one having been found and returned by a passer-by, the other complained that the N-ALIVE wallet was empty. One naloxone reply-card was received after 8^th^ December 2014 stating that naloxone had been used to save the life of a friend.

**Non-Fatal Overdose Admissions (NFOAs)**

Procedural changes within HSCIC have delayed our ability to report on NFOAs to Accident & Emergency.

**Table S8: Responses to Telephone Questionnaire**

| **Telephone Questionnaire** | **NOR** | **Control** | **Total** |
| --- | --- | --- | --- |
| **Number of Forms Completed**  - First Fortnight  - Second Fortnight | 36 (44%)  22  14 | 45 (55%)  21  24 | 81  43  38 |
| **Time to completion** (days)  - First Fortnight  - Second Fortnight |  |  | Mean 10, Sd. 3  Mean 22, Sd. 3 |
| **Told family member/friend about Naloxone**  (if randomized to Naloxone) | 86% (31/36) | NA |  |
| **Carriage rate of Naloxone**  (if randomized to Naloxone) | 64% (23/36) | NA |  |
| **How Often did you carry it?**  All  Most  Some | 47% (17/36)  14% (5/36)  3% (1/36) | NA |  |
| **Do you still have the Naloxone?**  Yes  No | 86% (31/36)  14% (5/36) | NA |  |
| If no, What did you do with the Naloxone?  Lost it  Gave it away  Saved other | 20% (1/5)  60% (3/5)  20% (1/5) | NA |  |
| **Heroin use** in the **past 3 days** after leaving prison  **(Smoke/inject)**  **(Inject, alone)** | 47% (17/36)  6% (2/36) | 31% (14/45)  18% (8/45) | 38% (31/81)  12% (10/81) |
| **Self-overdose<=2wks of release**  Overdose  Someone present  Naloxone given  Taken to hospital | 0  NA  NA  NA | 1% (1/43)  0  0  0 | 1% (1/81)  0  0  0 |
| **Self-overdose >2wks of release**  Overdose  Someone present  Naloxone given  Taken to hospital | 0  NA  NA  NA | 0  NA  NA  NA | 0  NA  NA  NA |
| **Presence at overdose of others**  **<=2wks of release**  Present  Naloxone given  Taken to hospital  Survived | 6% (2/36)  1/2  0/2  2/2 | 4% (2/45)  0/2  1/2  2/2 | 5% (4/81)  1/4  1/4  4/4 |
| **Presence at overdose of others**  **>2wks of release**  Present  Naloxone given  Taken to hospital  Survived | 0% (0/35)  Unknown  0  Unknown | 1% (1/45)  Unknown  1  Unknown | 1% (1/81)  Unknown  1  Unknown |
| **Naloxone acquisition-rate**  (if randomized to control) | 3% (1/36) | 2% (1/45) | 2% (2/82) |
| **Do you think taking part in N-ALIVE changed your own use of heroin in the first 2 weeks after release?** | | | |
| No  Safer Heroin Use  Riskier Heroin Use | 39% (14/36)  53% (19/36)  6% (2/36) | 40% (18/45)  49% (22/45)  2% (1/45) | 39% (32/81)  50% (41/81)  4% (3/81) |
